# Agreement Between Parental Report and Clinician Observation of Infant Developmental Skills

**DOI:** 10.3389/fpsyg.2021.734341

**Published:** 2021-11-02

**Authors:** Alexis Federico, Dexin Shi, Jessica Bradshaw

**Affiliations:** Department of Psychology, University of South Carolina, Columbia, SC, United States

**Keywords:** infant development, motor skills, social communication, assessment, parent report, interrater agreement, clinician observation

## Abstract

Understanding the convergence between parent report and clinician observation measures of development is important and became even more critical during the COVID-19 pandemic as clinician contact with families was significantly limited. Previous research points to inconsistencies in the degree of agreement between parents and clinicians and very little research has examined these associations for infants within the first year of life. This study investigated the association between parent report and clinician observation measures of social communication and motor skills in 27 young infants who were assessed at 9 and 12 months of age. Results suggest a strong relation between clinician and parent rated motor skills, but weak to moderate associations between clinician and parent rated communication skills. Infant temperament played a significant role in parent ratings of infant communication. Together, these results provide support for data collection via parent report or clinician observation of infant motor skills, but suggest that multiple measures of infant communication may be helpful to obtain high-quality, perhaps more accurate, assessment social-communication skills. Specifically, multiple parent report measures along with an observation of parent-infant interactions will likely provide a more rich and accurate characterization of infant social-communication abilities.

## Introduction

Mapping precise developmental change in infancy has resulted in significant theoretical and empirical advancements in developmental science (Oakes and Rakison, 2020; Iverson, 2021). The timing of emergence of new infant skills is a key component of these empirical pursuits and valid, accurate assessment of skills is critical. However, direct contact with infants is not always possible. During the COVID-19 pandemic, many developmental studies that relied on direct, clinician-administered measures of developmental skills were forced to transition to online, parent-report methods (Hunersen et al., 2021). Similarly, studies that collected only clinician-administered data, or both clinician observation and parent report data, transitioned to using parent report data only. This transition has highlighted the importance of understanding concordance between parent report and clinician observation measures of developmental skills. While multi-informant assessment of child skills has proven to be more valuable than single-informant methods in clinical settings (Li et al., 2019), the convergence and possible interchangeability of parent report and clinician observation developmental measures in infancy, especially within the first year of life, is not well understood. Moreover, knowledge of how parent report assessment of child skills converge with clinician observation assessments could lead to increasing popularity of online developmental studies, which will increase accessibility for underrepresented populations in research.

In research settings, assessments administered by expert clinicians are commonly used to characterize global developmental skills (Brito et al., 2019). Typically, these clinicians are highly trained with in-depth knowledge of assessment, development, and psychopathology. However, clinician assessments are not only expensive and time consuming, but can result in underestimation of skills due to other child-specific variables, such as inhibited temperament or child reluctance to interact with the examiner (c.f. Libertus and Landa, 2013). To combat these difficulties, many studies opt for parent report measures to evaluate infant developmental skills. These questionnaires provide unique access to parent’s knowledge about their children in a variety of contexts and over a longer period of time compared to clinician observation methods. Further, they are more cost-effective and less time intensive, and can be easily completed via online data collection methods. However, concerns about the validity of parent report questionnaires have been raised as ratings made by parents often do not correspond to ratings made by other professionals (Darling-Churchill and Lippman, 2016). In addition, several contextual family well-being factors have been found to affect parent report of child skills and behavior. Parents with higher ratings of depression often rate their child’s behavior as more problematic and challenging than parents with lower ratings of depression (Durbin and Wilson, 2012; Harvey et al., 2013). Parent education evidently plays a role in the ability to distinguish between receptive and expressive language skills, as defined by clinician-administered assessments, which could result in inflated scores (Reese and Read, 2000). Socioeconomic status has also been associated with the level of disagreement between parents and clinicians on measures of child language (Feldman et al., 2000; Meisenberg and Williams, 2008).

Studies that have directly investigated the association between clinician observation and parent report methods of data collection suggest that the degree of clinician-parent discrepancy is domain-specific. For example, Libertus and Landa (2013) found a strong association between parent report and clinician assessment of infant motor skills in the first 2 years of life. This finding is consistent with other literature documenting general parent-clinician agreement of child gross motor skills (Bodnarchuk and Eaton, 2004). In contrast, parent report of fine motor skills has shown greater disagreement with clinician assessment measures compared to other developmental domains (Scattone et al., 2011). In the language domain, stronger agreement for expressive language skills compared to receptive language skills has been observed (Luyster et al., 2008; Sachse and Von Suchodoletz, 2008). Luyster et al. (2008) found that for clinician observation methods, expressive language was rated higher than receptive language whereas parent report methods resulted in higher receptive compared to expressive language. The authors suggest that parents often over-estimate children’s verbal comprehension skills as it is challenging to parse apart differences between response to non-verbal (i.e., gestures) and verbal cues (Luyster et al., 2008). Similarly, Bennetts et al. (2016) found the strongest correlations between parents and clinician ratings of infant behavior to be in the expressive language domain for infants between 1 and 2 years. In contrast, Miller et al. (2017) found that parent report did not differ from clinician assessment in either domain of language.

Clearly the extant literature reflects inconsistencies in the association between parent report and clinician assessment methods for evaluating infant skills. This is especially important for developmental studies that cut across parent report and clinician observation methods of data collection. Moreover, research studies that collect parent report data via remote methods provide the greatest possible access to infants and families across the geographic and sociodemographic spectrum.

### Current Study

This study will add to the current literature on parent and clinician agreement by looking at infant skills at very young ages (i.e., 9 and 12 months), and comparing agreement over time. Few studies have evaluated how the association between parent report and clinician observation methods change over time in infancy, especially within the first year of life when these early skills are just emerging. This report aims to examine the association between clinician observation and parent report methods of data collection across two key developmental domains at 9 and 12 months of age: social-communication and motor skills.

## Materials and Methods

### Participants

A total of 43 assessments were conducted with 27 parent-infant dyads who were enrolled in a longitudinal study of infant development. Of the 27 infant participants, 48% were female (*n* = 13), 67% were white (*n* = 18), 22% were Black (*n* = 6), 4% were Asian (*n* = 1), and 7% were more than one race (*n* = 2). Participants were either at elevated likelihood (EL; *n* = 10) or low likelihood (LL; *n* = 17) for autism spectrum disorder (ASD) due to family history. Elevated likelihood infants had a full biological sibling diagnosed with ASD and low likelihood infants had no family history of ASD in first- or second-degree relatives. Diagnosis of the older sibling was confirmed through review of medical records that documented an ASD diagnosis by a licensed clinician using gold standard diagnostic measures and indication of ASD on two screeners (Rutter et al., 2003; Constantino and Gruber, 2007). Exclusion criteria for all participants included: gestational age < 37 weeks, congenital hearing or vision challenges, known genetic syndrome (e.g., Fragile X Syndrome, Down Syndrome), and significant pre- or perinatal complications requiring NICU stay > 5 days. EL and LL infants were analyzed together, but the effect of family history was explored by separating the groups in subsequent analyses. Due to limited sample size, potential differences between these groups are not the focus of the current study. All study procedures were reviewed and approved by University of South Carolina Institutional Review Board. Written informed consent to participate in this study was provided by the participants prior to beginning any study procedures.

### Measures

As part of the longitudinal study, infants visited the lab at 9 and 12 months of age. At each visit, parents first completed questionnaires about their infant’s social communication and motor skills. Subsequently, infants were administered assessments of social communication and motor skills by a licensed clinical psychologist with expertise in infant development or a doctoral student who was supervised by a licensed psychologist.

#### Parent-Report Measures

Child social-communication and motor skills were assessed via parent report at 9 and 12 months using the Communication and Symbolic Behavior Scales – Developmental Profile – Caregiver Questionnaire (CSBS-CQ; Wetherby and Prizant, 2003) and the Early Motor Questionnaire (EMQ; Libertus and Landa, 2013). The CSBS-CQ is a parent questionnaire that provides information on children’s language and social development. It results in an overall total score and three composite scores: Social, Speech, and Symbolic. The social composite measures eye contact, joint attention, and gestures, and includes questions such as “When your child is happy, does he/she smile or laugh and look at you at the same time?” The speech composite measures expressive verbal communication, and includes questions such as “Does your child use sounds to communicate both pleasure and discomfort?” Finally, the symbolic composite measures receptive language and play. Composite and total scores were calculated in accordance with the standardized manual for the CSBS-CQ (Wetherby and Prizant, 2003). The CSBS-CQ has strong test–retest reliability, with significant correlation coefficients above 0.64 for all composite scores and the total score (Wetherby et al., 2002). The CSBS-CQ also shows strong predictive validity of child language outcomes in young children (Wetherby et al., 2002).

The EMQ is a parent report measure of early fine and gross motor skills designed to match the motor scales of the Mullen Scales of Early Learning (Mullen, 1995) and other assessments of motor skills given by clinicians. Items on the EMQ are organized around different everyday situations and describe motor behaviors over the first 2 years of life. The EMQ has been validated against several clinician observation assessments, such as the Mullen Scales of Early Learning and Peabody Developmental Motor Scales, and has good concurrent and predictive validity (Libertus and Landa, 2013).

#### Clinician Assessments

Social communication and motor skills were assessed in the lab using the Communication and Symbolic Behavior Scales Developmental Profile Behavior Sample (CSBS-BS; Wetherby and Prizant, 2003), the Bayley Scales of Infant Development, 3rd Edition (BSID; Bayley, 2005), and the Mullen Scales of Early Learning (MSEL; Mullen, 1995). The CSBS-BS is a standardized assessment measure, administered by trained clinicians, created as a partner to the CSBS-CQ. It is designed to elicit communication from children in a natural, but controlled environment. Stimuli are presented to the child, for example a balloon that is blown up and then flattened, and then a trained examiner completes a series of steps to encourage the child to ask for help. The caregiver is present during this assessment and credit is given for child communication directed toward the caregiver or the examiner. The total score and composite scores are similar to the CSBS-CQ: social, speech, and symbolic. The social composite measures the child’s ability to look at a toy, to an examiner, and back to the toy as well as their ability to engage in joint attention initiated by the clinician. The speech composite measures the sounds and words the child produces. The symbolic composite measures the child’s ability to understand questions asked by the clinician and the child’s play skills. Total and composite scores were calculated according to the standardized manual for the CSBS-BS (Wetherby and Prizant, 2003). Examiners were trained to administration and scoring reliability by a gold standard (GS) examiner from the instrument author’s team. Administration reliability was defined by >80% of activities determined to be correctly administered by the GS examiner and scoring reliability was defined by >80% item-by-item inter-rater agreement with GS examiner. The CSBS-CS has also been shown to have strong test-retest reliability, concurrent validity, and predictive validity of later language outcomes (Wetherby et al., 2002; Wetherby and Prizant, 2003). Further, correlation coefficients were moderate to strong between the CSBS-CQ and CSBS-BS (*r* = 0.65−0.71) providing evidence for the concurrent validity of both the CSBS-BS and CSBS-CQ (Wetherby et al., 2002).

The BSID and MSEL are clinician administered assessments of motor, language, and cognitive development. The BSID was used to assess fine and gross motor skills at 9 months, and the MSEL was used to assess fine and gross motor skills at 12 months of age. The MSEL was administered at 12 months to be consistent with national studies of infant siblings of children with ASD; use of this measure across studies promotes data pooling and sharing (e.g., Ozonoff et al., 2011; Iverson et al., 2019). The BSID and MSEL are well validated, reliable, and widely used measures of infant motor development (Mullen, 1995; Bayley, 2005; Johnson and Marlow, 2006; Burns et al., 2013).

### Temperament

Infant temperament was assessed at each time point via the Infant Behavior Questionnaire – Revised (IBQ-R; Gartstein and Rothbart, 2003). The IBQ-R is a parent questionnaire that results in three subscales: surgency, negative affectivity, and regulation/orienting. The surgency scale measures positive emotionality, such as smiling and laughter and vocalizations. The negative affectivity scale measures negative emotions like fear and sadness. The regulation/orienting scale measures how well the child regulates their responses, including emotion regulation and soothability. The IBQR has excellent reliability and internal consistency with chronbach’s alphas above 0.70 (Gartstein and Rothbart, 2003). Moderate to strong inter-rater reliability has also been demonstrated with correlation coefficients falling between 0.30 and 0.70 (Gartstein and Rothbart, 2003).

### Data Analysis Plan

The primary aim of this study was to determine the association between parent report and clinician observed measures of behavior across social communication and motor domains in 9- and 12-month-old infants. Similar to Libertus and Landa (2013), we first used Pearson correlations to evaluate the strength and statistical significance of associations between parent and clinician measures. Infants were analyzed together within each time point, which allows us to examine how associations change over time. Standard scores are not available for the parent-report motor measure (EMQ) or the clinician-administered communication measure at 9 months (CSBS-BS) and so raw scores for all measures were used in all analyses. In addition, these associations were explored separately for EL and LL infants. Given the potential impact of temperament on child performance in the lab, especially with regard to social communication, the association between parent-reported infant temperament and parent-clinician discrepancy on social-communication measures was explored. Total social-communication discrepancy scores were calculated for each participant by subtracting the CSBS-BS Total raw score (clinician measure) from the CSBS-CG Total raw score (parent measure). Given the limitation of sample size, the strength of the association, indicated by the correlation coefficient, is emphasized and both correlation coefficients and *p*-values are reported.

## Results

**FIGURE 1:**
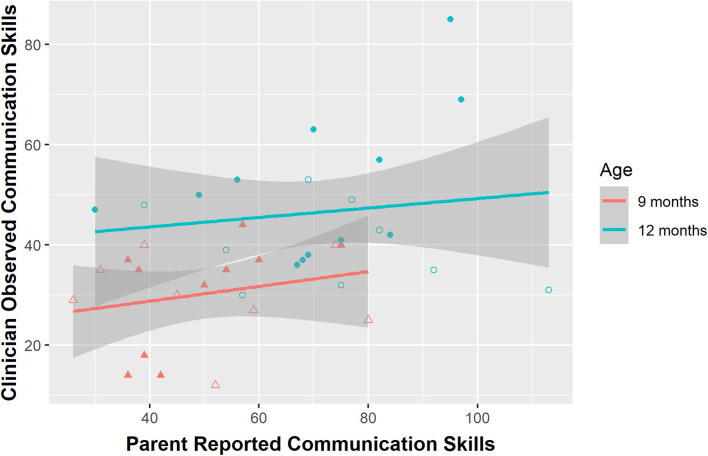
Association between parent report and clinician observation of social communication (raw scores) at 9 and 12 months. For illustrative purposes, elevated likelihood (EL) and low likelihood (LL) infants are depicted separately using open symbols for EL infants and closed symbols for LL infants.

Descriptive statistics and correlation coefficients across all parent report and clinician assessment measures are reported in Table 1. Upon visual examination, data appeared normal across both measures and age groups. At both 9 and 12 months, there was a weak, non-significant association between parent-reported and clinician-observed social-communication skills assessed using the CSBS-CG and the CSBS-BS (9 months: *r* = 0.246, *p* = 0.359; 12 months: *r* = 0.115, *p* = 0.629; see Figure 1). The strongest association across both time points was observed in the Speech composite at 12 months (*r* = 0.444, *p* = 0.052). Examination of CSBS standard scores at 12 months resulted in a negligible increase in the strength of clinician-parent agreement [*r* = 0.217, *p* = 0.359; *t*(46.91) = 1.40, *p* = 0.17] compared to raw scores and showed that in general, parent ratings resulted in higher standard scores compared to clinician ratings. In regard to motor skills, there was a strong association between 9-month parent-reported and clinician-administered gross motor (*r* = 0.636, *p* = 0.005) and fine motor skills (*r* = 0.545, *p* = 0.019). At 12 months, when the MSEL was used to evaluate motor skills, there was a very strong association for the gross motor domain (*r* = 0.702, *p* = 0.001), but not for the fine motor domain (*r* = −0.076, *p* = 0.772).

**TABLE 1:** Descriptive statistics and Pearson correlations for parent and clinician raw scores by domain.

	9 Months					12 Months				
		
	Parent average (SD)	Clinician average (SD)	Overall Pearson correlation	EL Pearson correlation	LL Pearson correlation	Parent average (SD)	Clinician average (SD)	Overall Pearson correlation	EL Pearson correlation	LL Pearson correlation
CSBS total	51.12 (15.63)	30.17 (9.23)	0.25	–0.12	0.60	72.30 (19.93)	46.95 (13.88)	0.12	–0.37	0.47
CSBS social	28.47 (7.31)	23.72 (6.22)	0.12	–0.12	0.44	35.80 (7.02)	33.90 (8.74)	0.14	–0.08	0.23
CSBS speech	10.94 (4.29)	3.11 (3.05)	0.18	–0.08	0.24	15.60 (6.10)	5.55 (4.77)	0.44^†^	0.27	0.70*
CSBS symbolic	11.71 (6.94)	3.33 (2.17)	0.18	0.36	0.25	20.90 (9.64)	7.50 (3.98)	0.15	0.02	0.36
Gross motor	−1.56(14.81)	36.00 (3.84)	0.64**	0.69	0.47	28.83 (17.24)	16.11 (2.42)	0.70**	0.76*	0.81**
Fine motor	−16.67(12.49)	24.35 (2.35)	0.55*	0.17	0.64*	−2.12(14.73)	16.95 (1.51)	–0.08	–0.32	0.07

Parent report and clinician observation measures are presented in raw scores and are not directly comparable. CSBS, Communication and Symbolic Behavior Scales. ^†^<0.1, *<0.05, **<0.01.

Correlation coefficients for EL and LL parent report and clinician observation assessment measures separately are reported in Table 1. At 9 and 12 months, weak, nonsignificant correlations were reported between parents of EL infants and clinicians in the social-communication domain (9 months: *r* = −0.12, *p* = 0.77; 12 months: *r* = −0.37, *p*-value = 0.33). Moderate correlations were reported between parents of LL infants and clinicians (9 months: *r* = 0.60, *p* = 0.07; 12 months: *r* = 0.47, *p* = 0.12). The only correlation to reach significance was the Speech composite score for LL infants at 12 months (*r* = 0.70, *p* = 0.01). In the motor skill domain, there was a moderate correlation between gross motor scores for EL infants at 9 months (*r* = 0.69, *p* = 0.09), and a strong correlation at 12 months (*r* = 0.76, *p* = 0.03). Weak correlations were found for the fine motor domain (9 months: *r* = 0.17, *p* = 0.72; 12 months: *r* = −0.32, *p* = 0.44). The parents of LL infants also had moderate to strong agreement in the gross motor domain (9 months: *r* = 0.47, *p* = 0.12; 12 months: *r* = 0.81, *p* < 0.01). There was a moderate association in the fine motor domain at 9 months (*r* = 0.64, *p* = 0.03) but not at 12 months (*r* = 0.07, *p* = 0.85).

Temperament factors of surgency, negative affectivity, and regulation/orienting were then associated with the parent-clinician social communication discrepancy at each age (see Table 2). A strong association was observed between social-communication discrepancy and surgency at 9 months (*r* = 0.633, *p* = 0.009) and 12 months (*r* = 0.680, *p* = 0.001) as well as regulation/orienting at 9 months (*r* = 0.462, *p* = 0.072) and 12 months (*r* = 0.537, *p* = 0.015). This pattern of results was similar when looking at the discrepancy between 12-month standard scores resulting from the parent and clinician CSBS measures (surgency: *r* = 0.728, *p* < 0.001; regulation/orienting: *r* = 0.670, *p* = 0.001). Parents who rated infants higher in surgency and regulation/orientation also exhibited a greater magnitude of difference (i.e., discrepancy) between parent and clinician rated social-communication skills. Moreover, surgency and regulation/orienting were highly associated with parent-report communication measures (surgency: *r* = 0.756, *p* < 0.001; regulation/orienting: *r* = 0.706, *p* = 0.001), but not clinician-administered communication measures (surgency: *r* = −0.039, *p* = 0.869; regulation/orienting: *r* = 0.126, *p* = 0.595).

**TABLE 2:** Temperament descriptive statistics and Pearson correlations with social-communication discrepancy.

	9 Months		12 Months	
		
	Score average (SD)	Pearson correlation	Score average (SD)	Pearson correlation
Surgency	4.94 (0.71)	0.63**	5.10 (0.75)	0.68**
Negative affectivity	3.59 (0.78)	0.10	3.74 (0.65)	0.04
Regulation/orienting	4.74 (0.48)	0.46	4.53 (0.87)	0.53*

*<0.05; **<0.01.

## Discussion

Precise understanding of agreement between parent report and clinician observation measures may be especially valuable for assessing communication in young, preverbal infants for which adult interpretation of infant behavior is a critical component. Overall, our results demonstrate that agreement between parents and clinicians was highly dependent on the specific developmental domain being assessed. Moderate to high agreement was observed for assessment of infant motor skills, but there was surprisingly low agreement among parent reported and clinician observed social-communication skills at both 9 and 12 months.

Agreement between parents and clinicians on assessments of infant gross motor skills was very strong, consistent with previous research (Libertus and Landa, 2013). Agreement was stronger at 12 months compared to 9 months, possibly because infant gross motor skills become more robust with age and therefore easier to assess over time. However, different instruments were used to measure motor skills at 9 and 12 months and this finding could also indicate that the EMQ is more aligned with motor scales on the MSEL compared to the BSID. Indeed, the gross motor section of the EMQ was created to parallel the MSEL (Libertus and Landa, 2013). The MSEL and BSID may have slight differences leading to a stronger association between the EMQ and MSEL than EMQ and BSID. Overall, parent report of 9- and 12-month gross motor skills is highly associated with clinician observation measures, providing support for the use of either parent report or clinician observation when a broad estimate of gross motor abilities is desired. In regard to fine motor skills, there was a moderate association between parent and clinician ratings at 9 months, but no relation at 12 months. Very few studies have investigated parent-clinician agreement of infant fine motor skills, but those that have suggest that fine motor skills have lower agreement compared to other domains (Scattone et al., 2011). The MSEL fine motor domain is heavily reliant on infant attention to the examiner and imitation skills while the EMQ relies on observation of spontaneous behavior in everyday settings. This difference may help to explain some of the parent-clinician discrepancy, but the lack of association remains surprising and warrants further study of exact discrepancies at the item level with a larger sample size.

Parent report and clinician observed measures of infant social-communication skills were not strongly associated at 9 or 12 months. The communication assessment used in this study, the CSBS-DP, was originally designed as a tiered screening and assessment tool that begins with a short screener (the Infant Toddler Checklist) and is intended to be followed up with both the parent report (CSBS-CQ) and assessment (CSBS-DP). The authors of the measure explicate that both parent report and direct observation measures be used together to obtain an accurate assessment of communication skills, as the CSBS-CQ alone is subject to parent over- or under-estimation of skills and the CSBS-BS is subject to underestimation of skills due to inhibited or uncooperative temperament (Wetherby and Prizant, 2003). This interpretation is consistent with our findings and a comparison of 12-month standard scores suggests that parents may over-estimate skills or that clinicians may under-estimate infant skills. Notably, the CSBS-BS places an emphasis on the infant’s *use* of communicative acts (i.e., gestures, eye gaze, sounds, and words) and only unequivocally communicative acts are scored. In contrast, the CSBS-CG inquires more generally about infant behavior and caregivers may have difficulty discerning communicative intent in very young infants.

Among the CSBS composite scores at 9 and 12 months, the strongest association was observed for the 12-month Speech composite. This is consistent with previous research showing that expressive language skills consistently elicit higher agreement than other social-communication skills (Wetherby and Prizant, 2003; Luyster et al., 2008; Sachse and Von Suchodoletz, 2008). Very few studies have investigated CSBS-BS and CSBS-CQ agreement in infants younger than 1 year of age and only relatively recently has the CSBS-BS been used to understand the development of communication in infants as young as 9 months (e.g., Bradshaw et al., in press). Thus, it is important that future studies continue to evaluate the use of the parent-reported and clinician-observed social communication measures in very young populations. It is possible that nonverbal communication skills may be difficult for parents to accurately report or may not be robust enough in such young infants to generalize from the home setting to the lab.

Parents generally had a much higher agreement with clinicians in the motor domain as compared to the social communication domain. This may be due to the interpretation required to answer questions about infant social communication at this age. Indeed, questions on the EMQ include “When lying on his/her tummy, your child will roll over to be on his/her back” whereas the CSBS-CQ includes questions such as “Does your child let you know that he/she does not want something that you are offering him/her?” The example question from the EMQ does not require any interpretation, just for the parent to indicate if the child rolls over or not. However, the example question from the CSBS-CQ requires that the parent correctly interpret their child’s communication to mean that they do not want something. This could be quite difficult considering the young age of infants in our sample. Our results suggest that studies using only parent report or only clinician observation measures should interpret their findings within this context, with the caveat that results may have limited generalizability. In order to explore the impact of group membership on the agreement between parents and clinicians, correlations were run separately for EL and LL groups. In the social communication domain, agreement between the parents of LL infants and clinicians was higher than agreement between the parents of EL infants and clinicians. This may be due to a number of individual factors; for example, infants with ASD have been shown to differ in prelinguistic vocal behaviors compared to LL infants (Paul et al., 2011). Thus, parents of infants at an elevated likelihood for ASD may have to engage in more interpretation of their child’s communicative behaviors than the parents of LL infants. In addition, parents of children with ASD may have vastly different experiences of infant and child development compared to parents of typically developing children, which may affect parent reports. However, sample size restrictions in this study prevent firm conclusions and generalizable results related to parent-clinician agreement among LL and EL infants. Thus, future studies may wish to evaluate this issue in a larger sample.

In order to determine possible explanatory factors for the low agreement observed between parent and clinician measures, particularly for social communication, we examined the effect of infant temperament. These results suggest a significant impact of temperament on the discrepancy between parent and clinician ratings of social communication, particularly for temperament constructs of surgency and regulation/orienting. Infants who score high in surgency generally express high excitement, positive anticipation of pleasurable activities, frequent vocalizations, enjoyment of novelty, as well as high levels of smiling, laughter, and motor activity. Regulation/orienting describes an infant who soothes with help from a caregiver, enjoys low-intensity activities, and sustains attention to objects. In the current study, infants who were higher in surgency and regulation/orienting demonstrated a greater magnitude of parent-clinician discrepancy (i.e., lower agreement). Moreover, surgency and regulation/orienting were highly associated with parent report of social communication, but not clinician observation, at both 9 and 12 months. While the relation between temperament and language development is well-established (Slomkowski et al., 1992; Laake and Bridgett, 2014), the pattern of associations here suggests that parents may misinterpret their infant’s overall positive affectivity and high vocal and motoric activity as verbal and nonverbal communication.

The pattern of parent-clinician agreement that was observed across developmental domains has possible implications for measurement of these skills. Motor assessments resulted in the highest agreement, suggesting that parent report may be a more efficient, cost-effective, less burdensome, and more accessible measure of infant motor skills. Compared to communication skills, motor skills may be easier to evaluate (i.e., is the action present or not) whereas social-communication skills tend to be more complex (e.g., determining communicative function of specific sounds and gestures) and affected by a number of contextual factors, including temperament. This interpretation would be consistent with our finding that temperament factors were associated with the degree of discrepancy between parent report and clinician observation of social communication.

There are a number of limitations that restrict the generalizability of our findings. The small sample size limited our power and future studies should investigate similar questions in a larger group of infants as well as compare infants across family context. Results of this study should be replicated in studies with a larger sample size. Future studies may also consider using more than one parent report and clinician observation measure to further understand the role of the rater in the type of data being collected. For example, use of the MacArthur Communication Development Inventories (Fenson et al., 2007) might provide additional insight into the discrepancy between parent-reported and clinician-observed communication skills. Parent interviews of child functioning, such as the Vineland Adaptive Behavior Scales (Sparrow et al., 2016), take into account parent report and clinical judgment and can be administered remotely. The Vineland has been shown to have stronger associations with clinician observation assessments than observed in this study (Scattone et al., 2011) and should be investigated as a complement to parent questionnaires when clinician observation assessments are not feasible. Different clinician observation measures were used to evaluate infant motor skills at 9 and 12 months of age, which may lead to differences in levels of agreement. Future studies should use one clinician observation measure to reduce this confound. This study also leveraged clinician observations that occurred in a laboratory environment which may contribute to the relationship between temperament constructs and agreement between parents and clinicians. Adding a group of infants who received a clinician observation within their home may provide further clarification in how temperament affects agreement between raters. The caregiver (e.g., mother, father, and grandmother) filling out questionnaires at each visit was not controlled for. Future studies may wish to assess differential agreement between mothers and fathers and clinicians. Evaluation of predictive relations between early measures and developmental outcomes was outside the scope of this study, but such an investigation is important in determining unique predictive utility of parent and clinician measures.

Overall, the results of this study show that parent report of motor skills is highly associated with clinician observation measures, but parent report of infant social-communication skills in the first year of life, when these skills are just emerging, is more complex. It will be important for research studies to incorporate multiple parent-report measures of social communication and language (e.g., Vineland and/or CDI) when clinician observation is not possible. In addition, a semi-structured, video-recorded parent-infant interaction that is later coded for social-communicative skills has proven to be reliable and predictive of developmental outcomes (Wetherby et al., 2016). This study adds to the current literature on agreement between parent report and clinician assessment measures by assessing patterns of agreement over time within the first year of life. Results of this study provide support for high agreement between parent report and clinician observation of motor skills, but lower agreement among social-communication assessments. These findings have implications for measure selection and, importantly, interpretation of findings using these measures. We suggest that when clinician observation of social communication is not possible, researchers should consider a combination of multiple parent report measures of communication and temperament, especially when evaluating communication in the first year of life. Further, results of studies using parent report as the sole measure for social communication should be interpreted within the context of individual and family factors that may effect parent report of infant skills, such as number of children in the house, parent mental state, and infant temperament. Notably, commonly used parent report measures are typically developed and validated with strong psychometrics, thus their use remains valid. However, a combination of measures that are interpreted within context will ensure that results of parent reported social communication skills are understood within the context of the child and their environment, both of which may affect parent ratings of infant social communication.

## Data Availability Statement

The raw data supporting the conclusions of this article will be made available by the authors, without undue reservation.

## Ethics Statement

The studies involving human participants were reviewed and approved by University of South Carolina Institutional Review Board. Written informed consent to participate in this study was provided by the participants’ legal guardian/next of kin.

## Author Contributions

AF organized the database. JB and DS contributed to the data analysis plan and performed the statistical analysis. AF and JB contributed to the conception and design of the study and wrote sections of the manuscript. All authors contributed to manuscript revision, read, and approved the submitted version.

## Conflict of Interest

The authors declare that the research was conducted in the absence of any commercial or financial relationships that could be construed as a potential conflict of interest.

## Publisher’s Note

All claims expressed in this article are solely those of the authors and do not necessarily represent those of their affiliated organizations, or those of the publisher, the editors and the reviewers. Any product that may be evaluated in this article, or claim that may be made by its manufacturer, is not guaranteed or endorsed by the publisher.
